# Management of acute upside-down stomach

**DOI:** 10.1186/1471-2482-13-55

**Published:** 2013-11-15

**Authors:** Tobias S Schiergens, Michael N Thomas, Thomas P Hüttl, Wolfgang E Thasler

**Affiliations:** 1 Department of Surgery, University of Munich, Campus Grosshadern, Munich, Germany; 2 Department of Surgery, Chirurgische Klinik München-Bogenhausen, Munich, Germany

**Keywords:** Upside-down stomach, Hiatal hernia, Paraesophageal hernia, Gastric incarceration, Gastric outlet obstruction, Gastric volvulus

## Abstract

**Background:**

Upside-down stomach (UDS) is characterized by herniation of the entire stomach or most gastric portions into the posterior mediastinum. Symptoms may vary heavily as they are related to reflux and mechanically impaired gastric emptying. UDS is associated with a risk of incarceration and volvulus development which both might be complicated by acute gastric outlet obstruction, advanced ischemia, gastric bleeding and perforation.

**Case presentation:**

A 32-year-old male presented with acute intolerant epigastralgia and anterior chest pain associated with acute onset of nausea and vomiting. He reported on a previous surgical intervention due to a hiatal hernia. Chest radiography and computer tomography showed an incarcerated UDS. After immediate esophago-gastroscopy, urgent laparoscopic reduction, repair with a 360° floppy Nissen fundoplication and insertion of a gradually absorbable GORE® BIO-A®-mesh was performed.

**Conclusion:**

Given the high risk of life-threatening complications of an incarcerated UDS as ischemia, gastric perforation or severe bleeding, emergent surgery is indicated. In stable patients with acute presentation of large paraesophageal hernia or UDS exhibiting acute mechanical gastric outlet obstruction, after esophago-gastroscopy laparoscopic reduction and hernia repair followed by an anti-reflux procedure is suggested. However, in cases of unstable patients open repair is the surgical method of choice. Here, we present an exceptionally challenging case of a young patient with a giant recurrent hiatal hernia becoming clinically manifest in an incarcerated UDS.

## Background

Upside-down stomach (UDS) is the rarest type of hiatal hernia (< 5%). It is characterized by herniation of the entire stomach or most gastric portions into the posterior mediastinum [1,2]. Both gastroesophageal junction and parts of the stomach migrate intrathoracically, thus UDS represents a large mixed type - sliding and paraesophageal (type 3) hernia [1-3]. By many authors, UDS is also referred to as type 4 hiatal hernia [4]. Other intra-abdominal organs can be involved in the herniation [5,6]. The pathophysiology of hiatal hernias remains poorly understood. Three pathogenic components are widely found in the literature which can individually exist in different proportions (1) increased intra-abdominal pressure (transdiaphragmatic pressure gradient); (2) esophageal shortening (fibrosis, vagal nerve stimulation); (3) widening of the diaphragmatic hiatus due to congenital or acquired structural changes of periesophageal ligaments and muscular crura of the hiatus [7]. The latter include abnormalities of elastin, collagens, and matrix metalloproteinases [7-10].

As hiatal and true paraesophageal hernia, UDS can manifest itself clinically in a wide variety of symptoms including substernal pain, heartburn, postprandial distress and fullness, dysphagia, postprandial nausea and vomiting [2,3]. They occur due to reflux related to the sliding component and mechanically impaired gastric emptying, thereby, the latter symptoms usually preponderate [4,11]. Chronic mucosal bleeding may cause anemia and is ascribed to venous obstruction of the migrated stomach [2]. While UDS itself is a very rare condition it is associated with a risk of incarceration as well as volvulus development. These complications can cause acute gastric outlet obstruction and thereby present clinically as acute abdomen. Further complications are acute and severe gastric bleeding, ischemia and perforation. All of these complications represent true emergencies as life-threatening conditions. Prevalence of acute symptoms or incarceration in paraesophageal hernia was reported to be 30,4% [12].

Once diagnosed, UDS should be surgically addressed by reduction of the migrated stomach, excision of hernia sac, and hiatal defect closure combined with an anti-reflux procedure as 360° or partial fundoplication. Laparoscopic repair provides benefits as reduced postoperative morbidity and hospital stay. Even if asymptomatic a surgical intervention is indicated as a conservative approach bears the risk of a high mortality rate due to complications which is significantly reduced by elective surgery [1,2,4,5,11]. In the light of only few series and cases reported, there is no clear evidence from review of the current literature for the management of acute paraesophageal hernia or UDS as very rare conditions [13]. In addition, there is an ongoing controversial discussion about whether prothetic reinforcement of the hiatus by mesh insertion is reasonable and effective. In the face of high recurrence rates several surgeons recommend the use of prosthetic meshes. However, many severe complications can be associated with mesh implantation as perforation necessitating partial esophagogastrectomy or acute erosive bleeding of the abdominal aorta [14]. In summary, there is still a considerable controversy regarding the routine mesh insertion and the quality of evidence is very low.

## Management of acute incarceration – case presentation

A 32-year-old male presented to the emergency department (ED) after acute thoraco-epigastric pain had set in after dinner several hours before. On arrival in the ED, his intolerant epigastralgia and anterior chest pain had been associated with acute onset of nausea and vomiting. The patient reported on having had recurrent substernal pain and dysphagia as well as mild symptoms of reflux which had persisted for more than a year. He reported on a previous surgical intervention due to a hiatal hernia, whereupon a anterior hemifundoplication had been performed two years ago. Furthermore the patient had a history of Ebstein’s anomaly which had been addressed by a reconstruction of the tricuspid valve a year ago.

**Figure 1:**
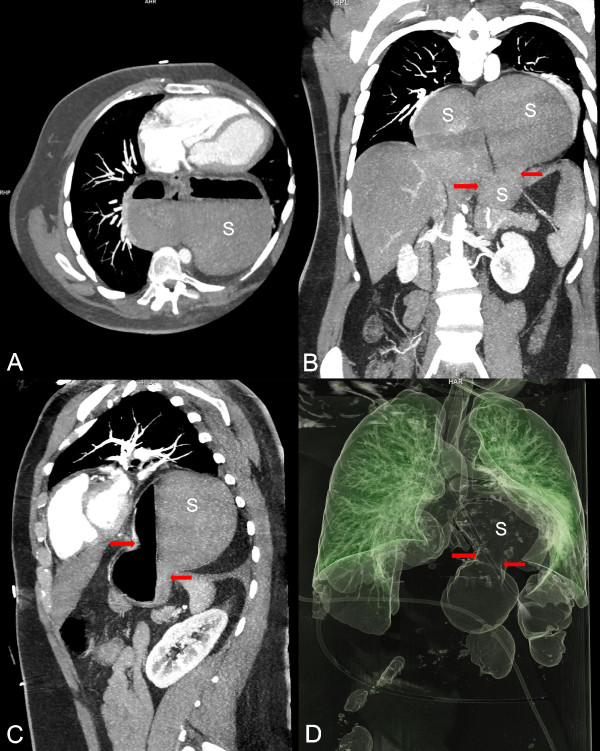
**Contrast-enhanced computer tomography. (A–C)** Giant mixed-type hernia (upside-down stomach (*S*)) with an incarcerated portion of the stomach (red arrows). **(D)** Visceral gas distribution seen from the *3D*-reconstruction showing the proximal gastric portion (S) in the posterior mediastinum (incarceration: red arrows).

**Figure 2:**
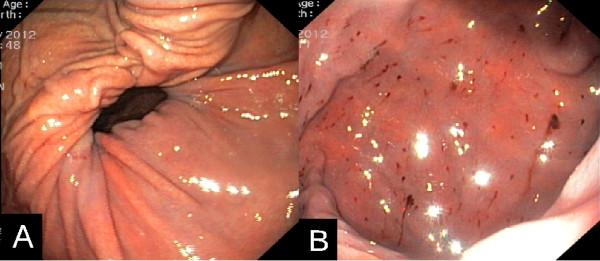
**Esophago-gastroscopy. (A)** Distended stomach migrated intrathoracically exhibiting the stenosis caused by the strangling diaphragm which could hardly be passed endoscopically. **(B)** Gastric mucosa appearing unremarkable aside from minor petechial bleedings.

A naso-gastric tube was tried to be placed but pushing it forward proved to be challenging and required repeated attempts, which all turned out to be unsuccessful. On admission the patient’s lactate level was mildly elevated (2.4 mmol/L) and besides a slightly increased WBC (12 / nL) unremarkable. Notably, no elevation of cardiac enzymes was detected. Electrocardiogram on admission showed sinus tachycardia, an incomplete right bundle branch block and a distinct S1Q3-pattern. Echocardiography revealed a normal left-ventricular ejection fraction, however the right ventricle was dilated. Upright chest radiography showed no subdiaphragmatic free air but visceral gas was seen in projection on the posterior mediastinum. Adjacent contrast-enhanced computer tomography disclosed a giant hiatal hernia (Figure 1). Most portions of the stomach and some of the greater omentum had migrated into the posterior mediastinum, whereas parts of the greater curvature appeared to be incarcerated in the diaphragmatic hiatus. Immediate esophago-gastroscopy showed a kinking-stenosis of the cardia and a stenosis caused by the strangling diaphragm which could hardly be passed. A naso-gastric tube was then positioned endoscopically and food residue and gas were sucked off for therapeutic decompression of the incarcerated stomach. Altogether mucosa appeared unremarkable and there were no signs of ischemia or restrained perfusion (Figure 2). After endoscopy the patient’s complains were attenuated but not resolved.

**Figure 3:**
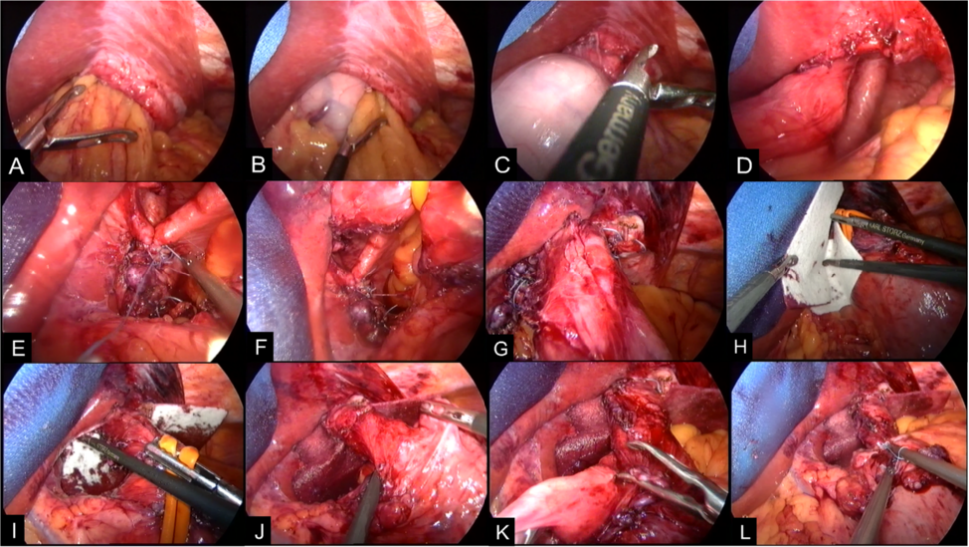
Laparoscopic reduction (A–D) and repair (E–G) of the incarcerated upside-down-stomach with insertion of a gradually absorbable mesh (H–I) and accomplishment of a 360° floppy Nissen fundoplication (J–L).

Emergent surgery for reduction of the incarcerated stomach and repair of the hiatal defect was performed through five trocars evenly dispersed to the upper abdomen (Figure 3). First, retracting the left liver lobe laparoscopic reduction of the stomach and attached portions of the greater omentum was conducted (Figure 3A–C) opening the view to a giant hiatal defect (Figure 3D). After preparation of the diaphragmatic crura and the distal esophagus preserving the rami of N. vagus a hiatoplasty was performed by anterior and posterior approximation of the diaphragmatic crura (Figure 3E–G). Given the fact of a recurrent hernia and a very wide defect of approimately 8 cm, a gradually absorbable GORE® BIO-A®-mesh (W.L. Gore & Associates Inc., Flagstaff, AZ) of biocompatible synthetic polymers was inserted enlacing the gastro-esophageal transition (Figure 3H–I). In a final step, a 360° floppy Nissen fundoplication was accomplished (Figure 3J–L). Postoperatively the patient recovered very well and was discharged five days later without any complication. He is to be followed up by the surgical outpatient department and is presently free of any complaints.

## Discussion

Surgery for incarcerated paraesophageal hernia or UDS has to be performed emergently as incarceration can become irreversible and severe bleeding can occur due to distension and vascular dilation. Moreover, ischemia and gastric perforation are on the verge. However, there are no clear evidence or existing guidelines on the management of acute paraesophageal hernia or UDS. Referring to this, Bawahab and colleagues have proposed algorithms based on the results of a series of 20 patients with acute presentation of paraesophageal hernia [13]. From this data and our experience, we suggest prompt open surgery in cases of unstable patients [4,13]. However, from our point of view, in case of gastric perforation or if there is any gastroscopic evidence of advanced gastric ischemia in stable patients, an initial laparoscopic approach is justifiable in case of adequate expertise, otherwise emergent open repair is suggested. In stable patients with acute presentation and mechanical gastric outlet obstruction due to incarceration as in the presented case, emergent laparoscopic reduction and repair is reasonable and prudent after urgent contrast-enhanced computer tomography and decompressing gastroscopy. For patients with acute presentation but without mechanical gastric obstruction and without gastric ischemia, we suggest a semi-elective repair. In summary, laparoscopic reduction and repair of acute paraesophageal hernia and UDS was shown to be safe in patients without gastric perforation or ischemia as well as feasible with low morbidity and mortality affording the benefits of minimally-invasive surgery [4,13]. Moreover, studies have been published reporting on percutaneous endoscopic gastrostomy (PEG) as useful and feasible approach [15-18]. Tabo *et al.* described a method facilitating the endoscopic reposition of the stomach by inserting a gastric balloon and to fixate the stomach subsequently applying the PEG-method (intraabdominal fixation of the stomach by gastrostomy) [18]. It may be an effective approach in elderly patients as the periprocedural risk is very low. In our young patient, however, we decided in favor of a laparoscopic approach repairing the hernia gate as sustainable therapy. In a series of 40 patients we could show that laparoscopic treatment of UDS is safe and highly effective using a laparoscopic hiatoplasty and anterior hemifundoplication [4].

As to the diagnosis in the ED, a high index of suspicion is essential when patients present acutely with epigastralgia and symptoms of upper gastrointestinal obstruction indicating mechanical gastric outlet obstruction. In our series, 5 of 50 patients with UDS (10%) presented with acute symptoms, two of them with gastric incarceration, one with upper gastrointestinal bleeding and one patient with omentum incarceration [4]. In another series of 147 patients, Allen and colleagues revealed that in 95% of all patients with UDS symptoms occurred which were primarily obstructive [11]. Complications of hiatal hernia are rarely considered in patients presenting with acute chest or epigastric pain as well as acute gastric outlet obstruction. Obstructive symptoms can range from mild nausea, bloating, postprandial fullness, dysphagia, retching or vomiting but rarely lead to the diagnosis in the ED. Hence, there is a high risk to mis- and underdiagnose an incarcerated UDS. Treatment as acute coronary syndrome (ACS) can have fatal consequences as gastric perforation [19,20]. Although information and sensitivity are low, plain chest radiography should be the first diagnostic tool whereby other differential diagnoses can be considered or ruled out. As a more reliable tool to work out the details of this important differential diagnosis contrast-enhanced thoracoabdominal computer tomography is suitable especially for the detection of complications as well as the decision for indicating surgery [19]. Impossibility of naso-gastric tube application as in our patient can be an evidence for gastric incarceration or volvulus as it is described by the *Borchardt’s Triad* consisting of the inability to pass a naso-gastric tube, usually unproductive retching as well as epigastric pain and distension [21]. The presented case shows the diagnostic challenge of acute presentation of paraesophageal hernia or UDS as they rarely feature one’s lists of differential diagnoses of acute epigastralgia or chest pain. Having confirmed the correct diagnosis, immediate decompressing esophago-gastroscopy and emergent surgery with reduction, hernia repair and antireflux procedure are able to prevent life-threatening complications.

## Conclusions

We present an exceptionally challenging case of a young patient with a history of Ebstein’s anomaly and a giant recurrent hiatal hernia becoming clinically manifest in an incarcerated UDS. In spite of anterior hemifundoplication two years ago the patient presented with this clinically and patho-anatomically impressive recrudescence. A genetically related common cause for cardiac and hiatal tissue defect can be hypothesized but was not assessed for lack of therapeutic consequences in this patient. However, given the fact of a recurring and very large hernia in spite of previous surgical repair as well as the postulated underlying tissue deficiency, we decided in favor of insertion of an absorbable mesh for hiatal reinforcement and tension-free repair. However, in the view of the above described complications associated with mesh implantation, we are exceedingly reserved regarding routine use of meshes and recommend thorough indication.

### Consent

Written informed consent was obtained from the patient for publication of this Case report and any accompanying images. A copy of the written consent is available for review by the Editor of this journal.

## Competing interests

The authors declare that they have no competing interests.

## Authors’ contributions

TSS and WET collected the patient’s history data. TSS drafted the manuscript with committed and dedicated review and discussion of MNT, TPH and WET. All authors contributed substantially to the patient’s care and therapy. All authors read and approved the final manuscript.

## Pre-publication history

The pre-publication history for this paper can be accessed here:

http://www.biomedcentral.com/1471-2482/13/55/prepub
